# The efficacy of green silica nanoparticles synthesized from rice straw in the management of *Callosobruchus maculatus* (Col., Bruchidae)

**DOI:** 10.1038/s41598-024-58856-4

**Published:** 2024-04-17

**Authors:** Samar Sayed Ibrahim, Huda Hassan Elbehery, Ahmed Samy

**Affiliations:** 1 Department of Pests and Plant Protection, National Research Centre, 33 El-Buhouth Street, Dokki, Giza, 12622 Cairo Egypt; 2 Department of Animal Production, National Research Centre, 33 El-Buhouth Street, Dokki, Giza, 12622 Cairo Egypt

**Keywords:** Biological techniques, Biophysics, Zoology, Nanoscience and technology

## Abstract

Rice straw, a byproduct of harvesting rice, must be disposed of by farmers in a variety of ways, including burning, which is hazardous for the environment. To address this issue, the straw needs to be utilized and turned into valuable products. One such product is nano-silica (SNPs), which will be synthesized and investigated in our study as a safe alternative to chemical insecticides. Rice straw-derived SNPs were synthesized using the Sol–Gel method. The contact toxicity of SNPs on *Callosobruchus maculatus*, a major pest of cowpea seeds, has been assessed. The size of synthesized SNPs was determined by transmission electron microscopy to be ~ 4 nm. The SNPs estimated LC_50_ on *C. maculatus* adults was 88.170 ppm after 48h exposure. By raising the tested concentration, SNPs treatment increased the mortality%, which reached 100% at 200 ppm exposures. Additionally, SNPs at LC_50_ treatment decreased adult longevity and the average number of emerged adults. The findings also verified that SNPs had no phytotoxic effects on the cowpea seeds germination. Rather, their application improved seed germination efficacy. This study proposed that rice straw can be utilized to manufacture highly efficient SNPs which can be efficiently employed to preserve stored grains from *C. maculatus* infestation.

## Introduction

More food is needed due to population growth; by 2050, food demand in developing nations is predicted to increase by 50% to 100%^1^. Pulses of the Fabaceae family are grown all over the world for their proteinaceous seeds. Their product could be used as low-cost animal feed as well^2–4^. Every year, stored grain pests damage 10–40% of grains in developing countries^5^. Many stored products as soybeans, red gram, lentil, cowpeas, and chickpeas, are severely harmed by the cowpea weevil, *Callosobruchus maculatus* F. (Col., Bruchidae)^6^. Seeds go through significant damage during storage, both qualitatively and quantitatively. The quantitative harm is caused by grain weight loss due to insect feeding, whereas the qualitative harm is caused by loss of nutritional value. The development of a single larva in a grain can cause weight losses of 8 to 22%^7,8^. Currently, conventional pesticides are the main method used to control *C. maculatus*. Pesticide resistance and pesticide residues are regarded as the most serious issues in pest control in stored grains. Integrated pest management programmes have implemented the use of effective and environmentally safe insecticides to prevent crop yield losses and increase agricultural productivity. Nanotechnology enables the development of new pesticides and insect repellants. However, the application of nanomaterials in agriculture, particularly for animal and plant production, remains an unexplored research area^9–11^. The diatomaceous earths (DEs), which are primarily composed of amorphous hydrated silica, is considered as one of the most thoroughly researched substitutes for chemical pesticides and have been evaluated by many researchers against numerous stored product pests. For instance, beetles^12–14^, moths^15^, and mites^16^. Aisvarya et al.^17^ and Debnath et al.^18^ reported that amorphous SiO_2_ NPs caused more than 90% and 100% mortality respectively, in *Sitophilus oryzae*. Arumugam et al.^19^ demonstrated the effectiveness of nanostructured silica as a control tool for *C. maculatus*. Because the industrial production of silica material using sodium silicate requires a large amount of energy^20^, it is critical to find another economically viable method of synthesizing nanosilica from biomass material. Rice straw is high in silica and a large capacity waste byproduct of the rice production at harvest. As a result, the synthesis of silica nanoparticles from rice straw aids in reducing waste and disposal issues. Rice straw is a valuable agricultural waste primarily composed of silica ash, lignin, and polysaccharides. The concentration of ash in rice straw is approximately 12.6 ± 0.11%, with 11.7% being silica. Rice straw has a higher silica content than hardwoods, softwoods, and other agro-residues. More than 80% of the silica in its ash can be extracted for possible uses^21,22^. According to the facts stated above, the present study aimed to investigate the insecticidal impact of silica nanoparticles synthesized from rice straw against *C. maculatus* adults, as well as the impacts of SNPs on certain biological parameters against adult insects. Furthermore, their impact on germination and growth of cowpea seeds was investigated.

## Results

### Silica nanoparticles

**Figure 1:**
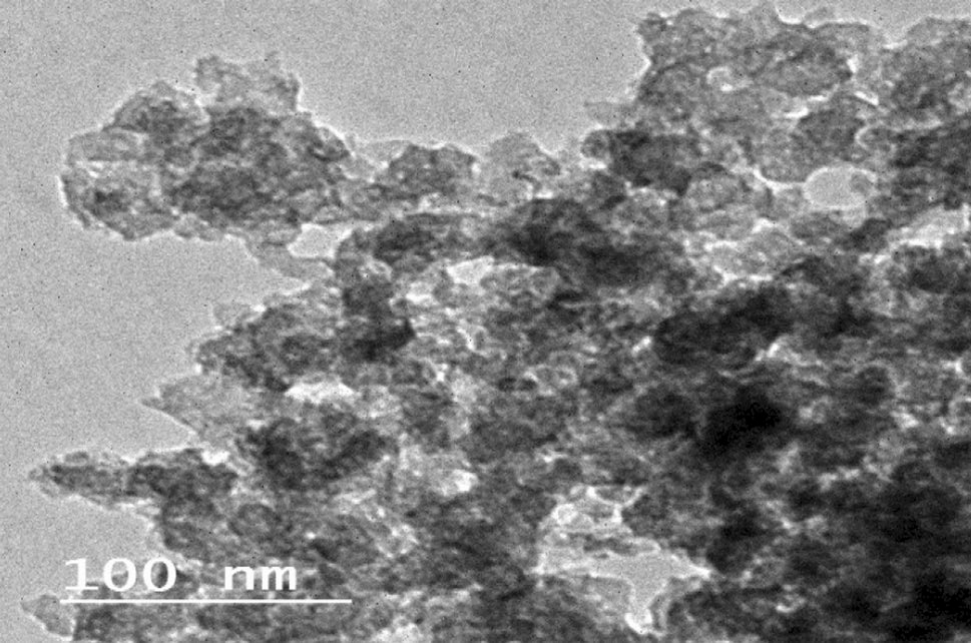
The nano silica image taken by a high-resolution transmission electron microscope.

Rice straw was utilized to manufacture nano-silica using an eco-friendly process, transforming harmful waste into economically valuable resources. Figure 1 illustrates that the nano-silicon dioxide appeared in a spherical form with around 4 nm an average size in the high-resolution transmission electron microscope picture.

### Toxicity bioassay

**Figure 2:**
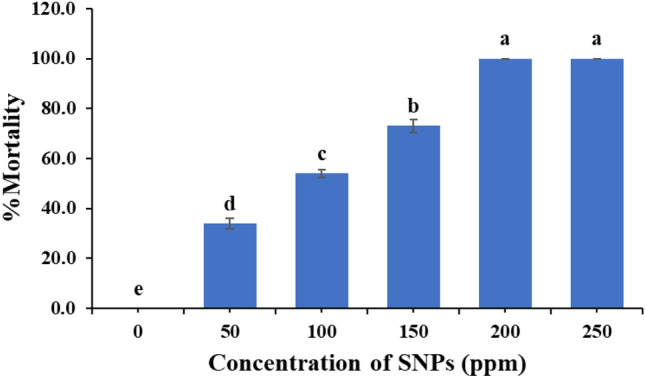
Effect of SNPs on mortality rate of *C. maculatus* after 48h exposure. Mean (± SE) values with similar letters are not significantly different (P < 0.05) (ANOVA, Duncan test).

**Figure 3:**
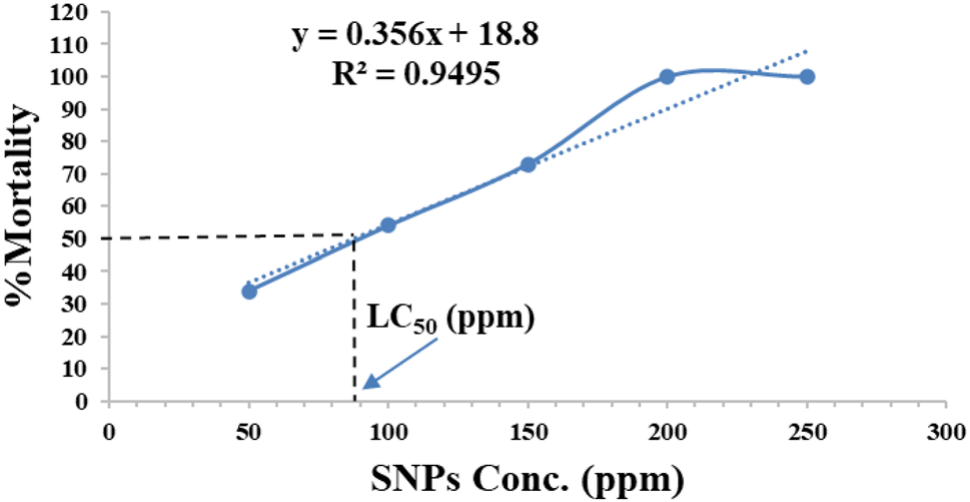
Graphical representation of SNPs concentration response curve for *C. maculatus* adults after 48h of exposure, based on Probit analysis with probability plot for linear regression and 95% confidence intervals.

Data obtained in Fig. 2 show that at highest concentrations of 250 and 200 ppm, *C. maculatus* adults’ treatment with SNPs caused 100% mortality after 48 h of treatment. The percentage of mortality decreased gradually by decreasing SNPs concentration. At dosages of 150, 100, and 50 ppm SNPs treatment caused mortality of 73.0 ± 2.60, 54.0 ± 1.63, and 34.0 ± 2.21%, respectively. The LC_50_ value for SNPs on *C. maculatus* adults after 48 h is shown in Table 1 and Fig. 3. It was noticed that percentage mortality increased by increasing the SNPs concentrations; the LC_50_ value for SNPs against *C. maculatus* adults was 88.170 ppm.

**Table 1:** Toxicity on *C. maculatus* adults treated with SNPs for 48h.

	LC_50_ (ppm)	95% confidence limits for concentration (lower–upper)	Chi^2^	Slope ± SE
SNPs	88.170	(77.047–97.804)	21.066	0.015 ± 0.001

### Biology bioassay

**Figure 4:**
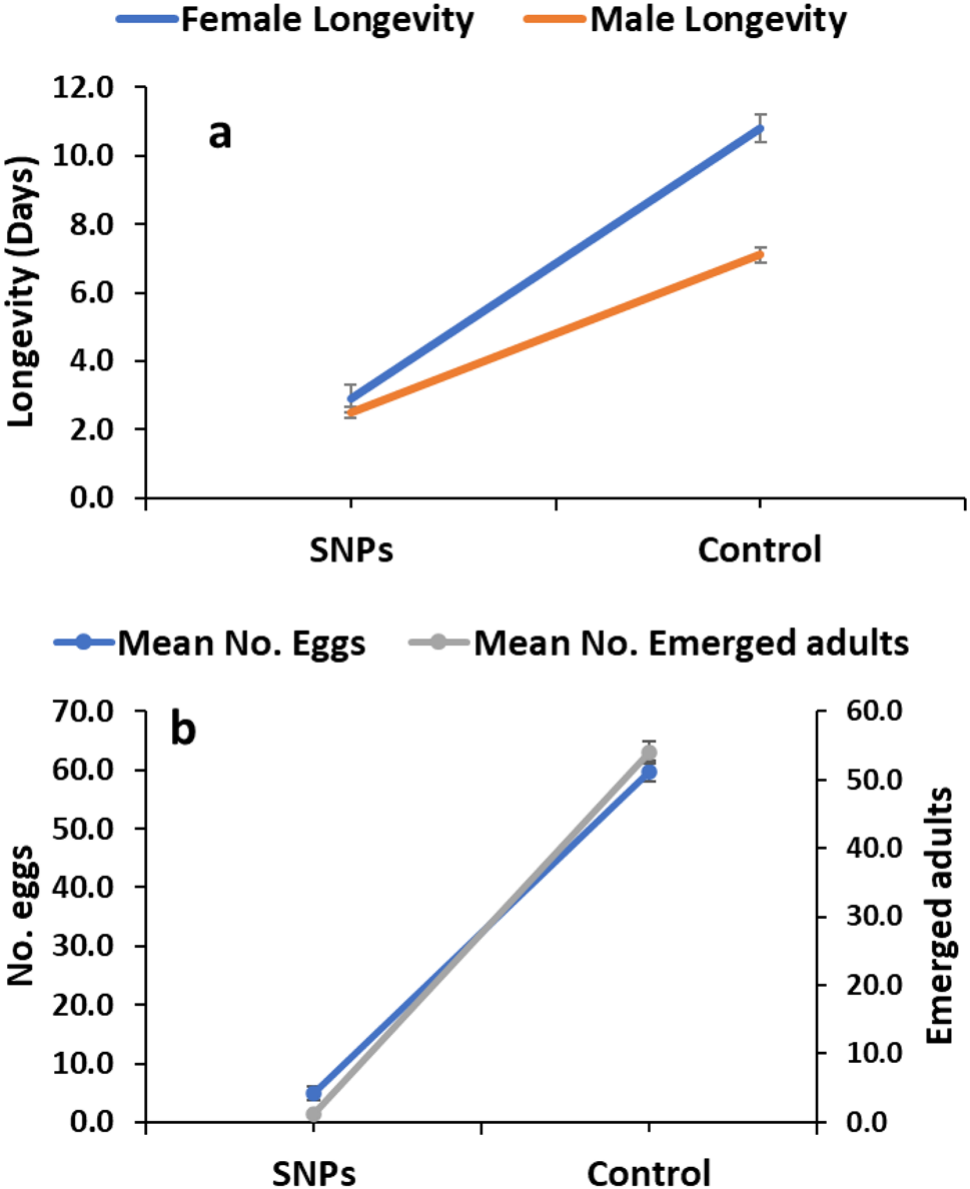
Effect of SNPs on *C. maculatus* adult longevity (**a**), and progeny production (**b**) after 48h of exposure.

The effect of SNPs treatment at LC_50_ (88.170) on biological development of *C. maculatus* adults was illustrated in Fig. 4. SNPs treatment significantly affected adult longevity; it was decreased in both females and males to 2.9 ± 0.4 and 2.5 ± 0.16 days, respectively, compared to the longevity of untreated female and male (10.8 ± 0.41 and 7.10 ± 0.23 days, respectively) (t: − 13.571, df,18, sig. 0.000; t: − 16.042, df,18, sig. 0.000) (Fig. 4a). The survived treated females laid significantly less number of eggs (4.91 ± 1.24 eggs/female) in comparison to that laid by untreated females (59.80 ± 1.71 eggs/female). Accordingly, the number of emerged adults was significantly decreased due to SNPs treatment; only 1.1 ± 0.27 of adults emerged in treated group while 54.10 ± 1.62 of adults emerged in untreated group (Fig. 4b).

### Seed germination

**Figure 5:**
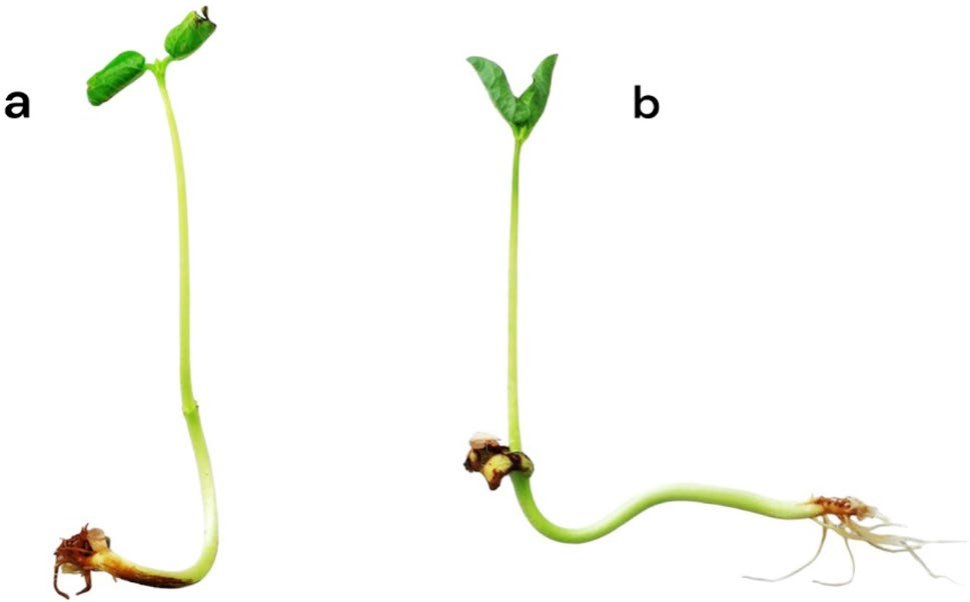
Effect of SNPs treatments on growth of cowpea seeds, (**a**) untreated seeds, (**b**) treated seeds.

Data in Table 2 revealed that germination parameters such as germination%, germination index, root length, and vigour index were increased but not significantly in cowpea seeds treated with SNPs by contact method for 48 h in comparison to untreated seeds (Fig. 5). A significant increase was only observed in the shoot length of treated seeds compared to untreated ones. The germination% in treated seeds reached 98% compared to 96% in control group. Also, the highest seedling vigour index recorded for treated seeds and reached to1752.80 with regard to1594.44 of control seeds.

**Table 2:** Effect of SNPs treatments on germination parameters of cowpea seeds.

	Germination%	Germination index	Length of the root	Length of the shoot	Vigour index
Control	96.00 ± 4.00^ ns^	1.00	2.01 ± 0.27^ ns^	14.56 ± 0.27*	1594.44 ± 85.39^ ns^
SNPs-treated seeds	98.00 ± 2.00^ ns^	1.02	2.22 ± 0.17^ ns^	15.64 ± 0.25*	1752.80 ± 51.03^ ns^
t	− 0.447	–	− 1.518	− 2.901	− 1.592
df (Sig. 2 tailed)	8(0.667)	–	8(0.167)	8(0.020)	8(0.150)

The Student's t test was used to compare means (P < 0.05).

*Significant.

^ns^not significant.

### Scanning electron microscope

**Figure 6:**
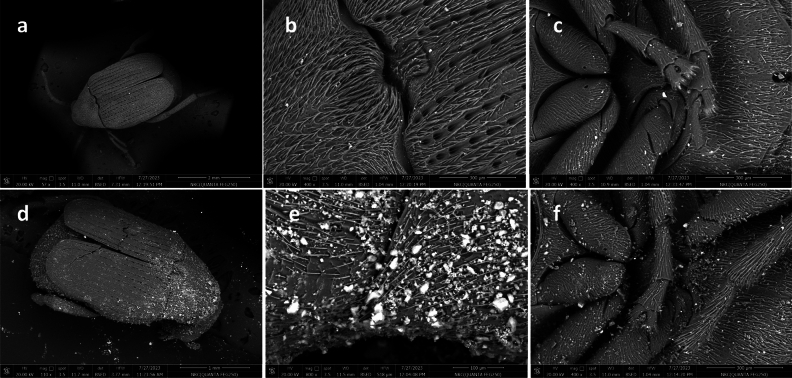
Scanning electron microscopic images of *C. maculatus* untreated adult dorsal view (**a**,**b**), ventral view (**c**), adult treated with silica NPs dorsal view (**a**,**b**) and ventral view (**c**), showing the scattering and attachment of nanoparticles on treated insect causing damage and abrasion.

**Figure 7:**
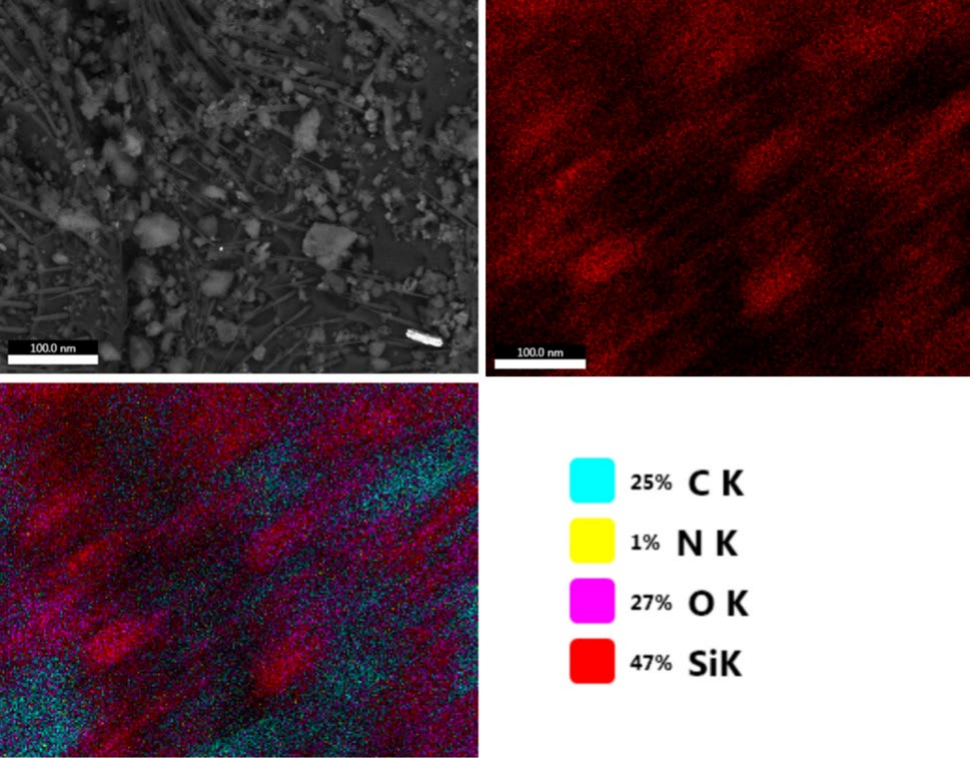
FESEM images of an insect's dorsal cuticle treated with 250 ppm SNPs reveal NPs impregnation and abrasion on the insect's body. Energy Dispersive Spectroscopy (EDS) measurements of silica (*Si*) indicate a high concentration of nanoparticles on the treated insect.

Scanning electron microscopic images of *C. maculatus* untreated adult are shown in Fig. 6a–c. SEM images of treated insects (Fig. 6d,e,f) revealed that SNPs are firmly attached to the surface of *C. maculatus* body as opposed to untreated insects. Adult *C. maculatus* appeared uniformly and densely NPs-coated when exposed to cowpea seeds treated with SNPs. The images also showed the strong adhesion of SNPs on both ventral and dorsal surfaces of *C. maculatus*, especially in its joints and intersegmental parts. In comparison to untreated adults, the NPs accumulated between the joints of the thorax and abdomen, along with the loss of insect legs, and attached in high density. Energy Dispersive Spectroscopy (EDS) counts of silica (*Si*) indicate a high degree of nanoparticles on treated insects, as shown in Fig. 7.

## Discussion

In this work, rice straw was used for synthesizing silica nanoparticles. Rice straw byproducts are obtained following the harvest, and the majority of farmers normally burn it before the next planting season, which subsequently pollutes the air. Silica nanoparticles were prepared in this study by Sol–Gel method. Our results confirmed the formation of round-shape particles with small nano size. The goal of the current study was to assess the entomotoxic properties of silica nanoparticles against *C. maculatus* as a potential substitute for the widespread application of chemical insecticides. The obtained results clarify that SNPs at very low concentrations severely affected the *C. maculatus* adults. A significant increase in mortality was observed at high concentrations, and even at lower concentrations, mortality percentages remained high to some extent. The results showed that the increasing SNPs concentration had increased the mortality of treated *C. maculatus* after 48 h. This was in line with data published by Salem^23^ and El-Bendary and El-Helaly^24^, who observed that mortality rate of insects increased by increasing concentrations and exposure times. The tested formulations of silica nanoparticles achieved significant effect against *Tribolium confusum, Rhythopertha dominica*, and C*. maculatus*^23^*.* In the same context, amorphous SNPs were highly efficient against *Sitophilus oryzae* and *Corcyra cephalonica* resulting in over 90% and 100% mortality, respectively, and an increase in exposure time and SNP concentration increased the mortality rate^18,25^. Ziaee and Ganji^26^ noticed that Aerosil and Nanosav silicon dioxide NPs are highly toxic to adults of *R. dominica* and *T. confusum*. Additionally, silica and silver NPs demonstrated 100% and 83% mortality rates against *C. maculatus* adults and larvae, respectively^27^.

Using the determined LC_50_ value, the effect of SNPs on certain biological attributes of adult *C. maculatus* was examined in the current study. It was observed that SNPs significantly reduced longevity of adult and number of deposited eggs/females, and subsequently the number of emerged adults was drastically lowered in treated group. Our findings were confirmed when compared to earlier investigations^28^ who reported that the highest concentration of silica NPs (2000 ppm) effectively reduced the number of deposited eggs in *C. maculatus* (9 eggs/ female) compared to control (98 egg/ female).This finding was consistent with earlier study^29^, who investigated the insecticidal activity of two silica forms (normal, 150 mm) and SNPs (35 nm) against *C. chinensis* and discovered that SNPs had more insecticidal activity, and at concentrations of 1.0 and 2.0 g NSPs /100 g broad bean seeds no adult insects emerged. Another study by Arumugam et al.^19^ agrees with results obtained in the present study, they tested the effect of hydrophobic SNPs on *C. maculatus* and reported a notably reduced egg deposition and adult emergence. The reduced fecundity of treated insects could be attributed to adults suffering from desiccation and spiracular blockage caused by SNPs, which may have prevented mating. Males often use their feet to attach themselves to the female's dorsal body during mating, and it seems that the grease that insects secrete on their body surfaces is crucial to this process. Lower concentrations of SNPs most likely resulted in incomplete mating, whereas higher concentrations prevent mating^30^.

It was declared that the size of synthesized particles is an important factor, with smaller particle size typically resulting in greater substance effectiveness. Particles of nanomaterials formed by metal oxidation, for example SNPs and alumina NPs, are electrically charged^31,32^, exhibiting a dipole–dipole interaction that facilitates aggregate formation^33^. Because of friction or contact with different surfaces, insects carry an electric charge that is produced by triboelectrification^34^. Hence, the ability of finely divided NP aggregates to firmly adhere to the body surface of insects through sorption increased due to their high specific surface area resulting from reduced particle size. The silicon dioxide content in addition to the reduced nano size range of the particles which raise the surface area to volume ratio, might be responsible for the high insecticidal potential of silica nanoparticles^26^. According to previous study, amorphous SNPs with an average size of 3 nm that were produced from rice husk using the Sol–Gel method had a highest specific surface^35^. Increased insects contact with particles due to a high surface-to-volume ratio resulted in more cuticle desiccation and death. Vayias et al.^36^ reported a significant positive correlation between the tested DEs' efficacy and the amount of small particles they contained.

Insects treated with SNPs most likely died from desiccation; specifically, nanomaterials cause cuticle destruction by physicosorption of lipids from the insect cuticle, causing the insect to lose water from its body and consequently die^37–40^. It was reported that the tested SNPs lowered the dry weight of cuticle, cuticular chitin, and total protein of *Agrotis ipsilon* after 24h^41^. This effect confirmed that dehydration was the primary cause of SNPs' ability to function as a nanocide. It was clearly observed that the dead *C. maculatus* insects due to SNPs treatment became dehydrated and shrunken; from SEM photograph it can be assumed that silica nanocide caused abrasion damage to the cuticle, with a small amount of adsorption damage as well. Similarly, *Plutella xylostella* larvae treated with SNPs (17–38 nm) died from desiccation, body wall abrasion, and spiracle blockage, according to light microscopy and SEM analysis^42^. Reduced particle sizes produced a very large surface area, a crucial factor that affects the potential of a substance for adsorption, which increases the amount of dust that comes into contact with the cuticle of insects^32,43^. Likewise, it has been reported that finely divided particles increase the insecticidal efficacy of dust^19^. They also reported that SNPs treatment resulted in nanoparticle adhesion throughout the insect body and elytra abrasion with noticeable scratches. In previous studies, SEM images showed that the very small size of alumina NPs (2–8 nm) and zinc oxide NPs (4 nm) boosted particle adhesion in the cuticle of *Sitophilus oryzae*, *Oryzaephilus surinamensis*, and *Sitotroga cerealella* adults^39,40^. Additionally, it was stated that even at low SNP concentrations, the exposed seed surfaces that could interact with insects increased significantly when the particle size decreased (12 nm), enhancing the insecticidal efficacy of SNP^19^. In brief, the following might explain some of the mechanisms by which inert dusts, such as SNPs, function: They absorb water from the insect's cuticle, block the spiracles of the insect, scratch the cuticular layer by settling between the segments and cause abrasion, and lead to excessive water loss by absorbing the lipids present in the waxy layer. According to Smith^44^, insect mortality triggered by dust particles may be related to digestive tract dysfunction or surface integument enlargement brought on by dehydration or tracheal and spiracle blockage. Both sorption and abrasion can harm the insects' cuticle-mounted protective wax covering.

Results about the toxicity of SNPs on the germination and growth of cowpea seeds, confirmed that SNPs had no phytotoxic effect. On the contrary, the application of silica nanoparticles increased the potential of the seeds through enhancing the efficacy of germination and the quality of seedling. Our findings are consistent with research by Hasanaklou et al.^45^ who showed that seed priming with nSiO_2_ (I) at 10 ppm was more efficient at enhancing germination rate, root and shoot dry weight, and germination percentage. Similarly, these results supported by Abdou et al.^46^ who reported that silica nanoparticles improved the germination and growth of pulse seed of the three varieties (chickpea, cowpea, and kidney bean). Furthermore, the development and germination of seeds from six different types of pulses treated with SNPs were unaffected^19^. Likewise, Alsaeedi et al.^47^ reported that all seedlings treated with SNPs had improved and higher germination characteristics and indices. Chourasiya et al.^48^ found that the percentage of seeds that germinated, the length of the shoots and roots, and the size of the seedlings were all significantly improved by nanopriming with nano-silica and nano-silica loaded with GA3. Recently, many studies supported applying nanosilica as fertilizer for crops where silica nanoparticles boosted the seed germination by their direct and indirect contribution in the growth of root and shoot^49,50^. The United States Department of Agriculture (USDA) has approved the use of amorphous silica as safe, and the International Agency for Research on Cancer (IARC) states that it is not carcinogenic^51^. According to a recent study, the addition of silicon dioxide nanoparticles synthesized from sand or rice straw's ash to broiler diets has the potential to enhance production, bone properties, and antioxidant status. This improvement was observed without any negative effects on blood biochemical parameters, liver function, or renal function^52^. Additionally, in a study conducted by Burton et al.^53^, it was found that the inclusion of silica at a concentration of 1000 ppm in the broiler feed resulted in enhanced weight gain and improved bone strength while maintaining the same bone mineral content. Previous research examined the cellular toxicity of SNPs in human fibroblast cell lines and the acute oral toxicity nanoparticles in mice^54^. Their findings suggested that nanosized materials are generally non-toxic. Since amorphous silica is comparatively biosafe, it might serve as a safer substitute for traditional toxic pesticides. To address the safety concerns of SNPs on non-target organisms and human health, more research is needed.

## Conclusions

It is obvious from our results that rice straw, which is considered a serious disposal problem as a waste material can be transformed to be a natural source for SNPs. The synthesized SNPs can be used safely as nanocide to help in the protection of stored products from *C. maculatus* damage. The synthesized SNPs from rice straw exhibited a toxic action on *C. maculatus*, and significantly affected the biological parameters of treated insects. Additionally, the maximum concentration tested of SNPs did not harm the treated cowpea seeds. It can be concluded that amorphous nano-silica has been successfully synthesized from rice straw and it displayed promising potential as a green insecticide. However, more research is needed to study the possible implications of the use of SNPs for agricultural purposes.

## Materials and methods

### Preparation and characterization of silica nanoparticles

The nano-silica material utilized in this investigation was synthesized from rice straw using the Sol–Gel method, involving the refluxing of rice straw ash with sodium hydroxide (3N) followed by neutralization of the product with HCL (3N), according to Samy et al.^32^. This environmentally conscious approach effectively transforms waste that poses environmental concerns into a highly efficient material.

### Insect

For multiple generations, *Callosobruchus maculatus* was maintained on cowpea seeds in glass jars (10 × 9 × 18 cm) with fine muslin cloth covering them for ventilation in a controlled laboratory environment (30 ± 2 °C, 65 ± 5% relative humidity). *C. maculatus* adults used in experiments were 1–3 days old. Clean cowpea seeds were purchased from the local market to use as food for insects and for experiments. The seeds were sterilized prior to use by being cold stored at − 18 °C for a minimum of five days. Rearing insects and experiments were carried out in the National Research Center's Pests and Plant Protection Department laboratory.

### Toxicity bioassay

To examine the toxicity of SNPs, adult insects of *C. maculatus* were used for contact/feeding toxicity bioassay. Ten replicates containing ten adults and forty grammes of cowpea seeds were used for each treatment, in addition to the control group. Cowpea seeds were mixed with SNPs dust in small plastic screw-capped jars (120 ml) at concentrations of 50, 100, 150, 200, and 250 ppm, in the rate of 2, 4, 6, 8, and 10 mg/40 gm of seeds. To achieve an even distribution of nanoparticles on cowpea seed, for two minutes the jars were handily shaken. Another group served as a control, receiving only cowpea seeds (no SNPs). Throughout the duration of the 48-h experiment, the number of *C. maculatus* insects that were alive and dead were recorded, and the concentration-mortality percentage was noted. After being prodded with a fine brush, insects were deemed dead if no leg or antenna movements were observed.

### Biology bioassay

The insecticidal efficacy of SNPs on some developmental parameters of *C. maculatus* was determined using LC_50_ (ppm) of SNPs. Forty grammes of SNP-treated cowpea seeds were put into plastic screw-capped jars. Each jar also contained a pair of freshly emerged *C. maculatus* adults. Ten replicates of the experiment were conducted; as a control, another set of paired adults received cowpea seed that had not been treated with SNPs. On a daily basis, the treated and untreated groups were observed for recording the duration that the males and females lived, to count the number of eggs a single female laid on the seeds, and to compute the percentage of adult emergence.

### Effect of SNPs on cowpea seeds

To investigate the viability and germination percentage of treated cowpea seeds, SNPs were used at the highest tested concentration i.e., 250 ppm. Inside a petri dish (11 cm), treated seeds were distributed on a piece of moist cotton. Five replicates of the experiment, each containing ten seeds, were carried out. Another five replicates were kept for untreated seeds as control group, and all groups were kept at room temperature. The germination count was recorded for eight days after sowing, the number of normal seedlings was expressed as a percentage of germination, and the final germination count was recorded in accordance with International Seed Testing Association^55^ regulations. The following equations were used:

$$ GI = \frac{\% Gt}{{\% Gc}} $$

GI: germination index; %Gt: % germination in treatment; %Gc: % germination in control.

In order to calculate the vigour index of seedling (VI) for the treatment and control groups, the lengths of the shoots and roots were measured after eight days. The VI was then calculated using the following formula^55,56^.

$$ VI = \left( {AvRL + AvSL} \right) \times \% G $$

VI: Seedling vigour index; AvRL: Average root length; AvSL: Average shoot length; %G: % seed germination.

### Scanning electron microscopy

Field Emission Scanning Electron Microscopy (FESEM) was utilized to examine the adhesion of synthesized SNPs to *C. maculatus* adults, and for mapping the insect body's *Si* count. The dead insects previously treated with SNPs highest concentration (250 ppm) were collected and air-dried for approximately 5 days before examination. Then, adult insects were viewed under low vacuum SEM (TESCAN, Vega III/Czech Republic), in the Electronic Microscope Unite, Central Laboratory, National Research Centre.

### Data analysis

LC_50_ values determined by the Probit analysis method^57^ using SPSS software. The ANOVA Duncan test was used to analyze mortality data, and independent-Samples T test was used to analyze collected data from biological and seed germination experiments (SPSS, Version 18.0).

## Data Availability

The datasets used and/or analyzed during the current study are available from the corresponding author on reasonable request.
